# Differential scanning calorimetric study of antibiotic distamycin A binding with chromatin within isolated rat liver nuclei

**DOI:** 10.1080/13880209.2016.1258427

**Published:** 2016-12-16

**Authors:** Andrey N. Prusov, Galina Ya. Kolomijtseva, Tatiana A. Smirnova

**Affiliations:** a A.N. Belozersky Institute of Physico-Chemical Biology, M.V. Lomonosov Moscow State University, Moscow, Russia;; b All-Russia Research Institute of Agricultural Biotechnology, Russian Academy of Sciences, Moscow, Russia

**Keywords:** Nuclei, chromatin structure, differential scanning calorimetry, distamycin A

## Abstract

**Context:** Natural oligopeptide antibiotic distamycin A (Dst) biosynthesized by *Streptomyces distallicus* is traditionally used in medical practice as an anti-inflammatory and antitumour drug.

**Objective:** Dst was investigated for its effect on the structural components of native chromatin directly within isolated rat liver nuclei in the presence of physiologically significant cations (magnesium or spermine and spermidine).

**Materials and methods:** Differential scanning calorimetry (DSC) was used to study the Dst action at molar ratio Dst/DNA = 0.1 and 0.15 mM Dst on the melting profile of nuclei suspension in different conditions.

**Results:** Results showed that the thermodynamic parameters of control nuclei in the presence of polyamines or Mg^2+ ^were different. The incubation of nuclei with Dst raised transition temperatures of relaxed (peak II) and topologically constrained DNA (peak III) by 6–8 °C and decreased by 2–4 °C that of core-histones (peak I). The total excess transition enthalpy (Δ*H*_exc_) in buffer with polyamines (24.7 kJ/mol DNA nucleotides) increased by1.5 times versus control but in buffer with Mg^2+^, the value of Δ*H*_exc_ (35.8 kJ/mol DNA nucleotides) remained unchanged.

**Conclusions:** The association of Dst with chromatin in the nucleus weakens histone-DNA contacts and causes additional strengthening of interaction between two complementary DNA chains. Our results contribute towards validation of DSC to test drug ability to modulate chromatin structure in the physiological environment and to clarify the mechanism of these modulations.

## Introduction

Distamycin A is a natural oligopeptide antibiotic biosynthesized by *Streptomyces distallicus.* As an anti-inflammatory and antitumor agent, it is widely used in medical practice. Dst is known to bind selectively at AT-rich sites in the minor groove of DNA and to affect thereby many biological processes (Chaires 1998). The binding mechanism of Dst with DNA and the thermodynamic parameters of this association have been widely studied. It should be kept in the mind that the physiological substrate for Dst is not a naked DNA but the complex of DNA with proteins – chromatin, having changed compactness across cell cycle and located in the cell nucleus with volume ∼500 μ^3^. The special features of nuclear chromatin are the high concentration of DNA (∼20–40 mg/mL) and the existence of a macromolecular ‘background’ environment, creating the so-called crowding effect (Schnell & Hancock 2008). At the crowding conditions, the interaction of ligand molecules with their complementary sites may differ from that in a solution and may not obey the classical kinetics. The apparent binding constants may exceed those in dilute solutions by as much as several orders of magnitude. Thus, the screening of drugs for the pharmacological activity should take place under conditions that mimic the crowding effect of the physiological medium in which the ligand binding would actually occur.

According to the current paradigm, a structural unit of nuclear chromatin is the 10 nm beads-on-a-string fibril (nucleosomes fibril) (Gilbert et al. 2005). Nucleosome consists of the 147 bp DNA stretch wrapped 1.75 times around an octamer of core histone proteins H2A, H2b, H3 and H4 forming the core-particle, connected by a linker of variable length DNA and histone H1 attached (Kornberg 1974). Further packing of nucleosomes fibrils in the higher order structures remains unclear. The conception of hierarchical packing of chromatin by further twist and coil until chromosomal-level compaction (Belmont & Bruce 1994) is now supplemented by the conception of ‘polymer melt’ state of chromatin (Maeshima et al. 2010). Both these possibilities may really occur at the high nucleosome concentrations *in vivo*. It has been demonstrated that the complex 3D-structure of nuclear chromatin from the thermodynamic point of view may be described as a sum of energetically independent domains specific for different cell types, cell cycle stages, metabolic activity and physiological environment (Touchette et al. 1986; Rice et al. 1988).

A differential scanning calorimetry (DSC) is an informative biophysical method for the study of the molecular interactions inside the nucleus. In spite of the complex composition of the nucleus, its DSC thermogram is relatively simple and very sensitive way to investigate different actions on nuclei. Therefore, this method may be potentially used to test the effects of the anticancer drugs on nuclear chromatin and to distinguish between their mechanisms of action.

We report here the application of the DSC method to study the thermodynamic aspects of Dst binding with chromatin DNA within isolated rat liver nuclei.

## Materials and methods

### Isolation of hepatocyte nuclei

The nuclei were isolated from the liver of outbred white female rats (100–150 g body weight) as described previously (Prusov et al. 2010, 2015). The Animal Ethics Committee of the Institute approved the study protocol. Liver was homogenized in buffer A (15 mM TEA-HCl, pH 7.6, 80 mM KCl, 2 mM EDTA, pH 7.0, 0.2 mM spermine, 0.5 mM spermidine) or in buffer containing 20 mM TEA-HCl pH 7.6, 30 mM NaCl, 10 mM MgCl_2_ in 8% sucrose. To suppress protease and nuclease activities these solutions immediately before homogenization and all other solutions used for nuclei isolation were supplemented with phenylmethylsulphonylfluoride (Sigma, St. Louis, MO) to a final concentration of 0.2 mM and *N*-ethylmaleimide to 4 mM (Sigma, St. Louis, MO) (Cain et al. 1995). The homogenates were mixed with a solution of 2.5 M sucrose in appropriate homogenization buffer up to a final concentration of sucrose 2.1 M and centrifuged at 50,000 *g* for 45 min. The nuclear pellets were resuspended in appropriate buffer without sucrose and pelleted for 5 min at 1500 *g*. The concentration of DNA and RNA in the nuclei suspension was determined spectrophotometrically (Spirin 1958). The isolated nuclei were stored at −60 °C in 60% glycerol. Before using, the nuclei were washed from glycerol by buffer A or B (20 mM TEA-HCl pH 7.6, 5 mM MgCl_2_) and suspended in these buffers at concentration 1.5 mM of nucleotides.

Distamycin A (Sigma, St. Louis, MO) was dissolved in distilled water and stored at −20 °C for 3 days. The concentration was estimated using molar extinction coefficient of 34,000 M^−1 ^cm^−1^ at 303 nm (Dasgupta et al. 1987). Dst was added to the final concentration 0.015–0.15 mM and nuclei suspensions were incubated at room temperature for 30 min prior to calorimetric scanning.

### The differential scanning calorimetry (DSC) of nuclei suspensions

The thermal denaturation of nuclei was investigated by DSC using a DASM-4 microcalorimeter (Biopribor, Pushchino, Russia) with 0.47 mL capillary platinum cells. All experiments were carried out at a heating rate of 2°/min in the temperature range from 25 to 120 °C and at a constant pressure of 3 atm. The second heating was used as the instrument baseline because of irreversible denaturation found. The chemical baseline was calculated and subtracted using Origin 1.16 software (MicroCal. Inc., Malvern, UK).

## Results

We used two standard methods of nuclei isolation: in buffer A with polyamines or in B with Mg^2+ ^ions, which preserve the compact chromatin state similar to that in rat liver nuclei *in situ*. The electron microscopic images of nuclei purified by centrifugation through high-density sucrose in both buffer systems demonstrate nuclear chromatin as globules with a diameter of 100–200 nm, cytoplasmic contaminations were absent (Prusov et al. 2010, 2015).

**Figure 1.**
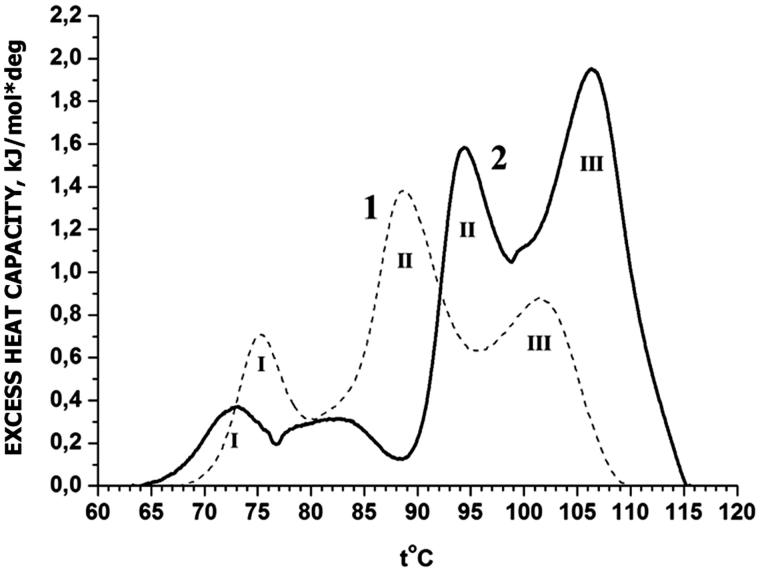
DSC profiles of rat liver nuclei in buffer A: 1 – control; 2 – with Dst (molar ratio Dst/DNA =0.1). I, II, III – designations of thermal transitions.

**Figure 2.**
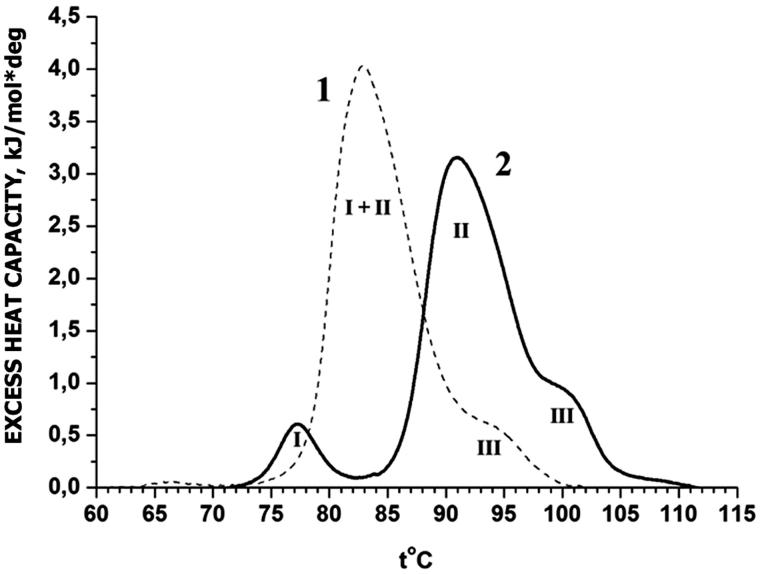
DSC profiles of rat liver nuclei in buffer B: 1 – control; 2 – with Dst (molar ratio Dst/DNA =0.1). I, II, III – designations of thermal transitions.

The melting of rat liver nuclei in buffers A and B are shown in Figures 1 and 2 (curves 1). DSC profile of these rat liver nuclei does not have peaks in the range from 25 to 65 °C attributed to the nonchromatin components (Balbi et al. 1989). Nuclear thermogram in buffer A reveals three well-separated peaks at Tm (transition temperature) of 75, 89 and 101 °C marked by Roman numerals as peaks I, II, III. Identification of peaks on DSC-thermograms continues to be a subject of discussions. The thermal transition at ∼75 °C and physiological ionic strength has been proposed as denaturation of the linker DNA domain (Balbi et al. 1989) or core-histones (Almagor & Cole 1989a). Peaks higher than 75 °C describe the melting of DNA: peak II – DNA from nucleosomal unfolding chromatin loops (Balbi et al. 1989) or relaxed DNA (Almagor & Cole 1989a) and peak III – melting of DNA from core-particles within condensed domains (Balbi et al. 1989) or topologically constrained DNA (Almagor & Cole 1989a). According to our preliminary data, the existence of these peaks does not depend on the condensation of chromatin in the nucleus. Therefore, we preferred to use the identification of M. Almagor and D. Cole. On our thermograms, peak I represent denaturation of core-histones, peak II that of relaxed DNA and peak III of topologically constrained DNA.

On the profile of the thermogram of nuclei in buffer B, one may see only the main peak at 83 °C and a pronounced shoulder at 93 °C. The values of Δ*H*_exc_ for nuclei in buffers A and B were different (24.7 and 35.8 kJ/mol nucleotides of DNA, respectively). The Δ*H*_exc_ values of each peak are represented in the Table 1.

**Table 1.** Enthalpies of thermal transitions for rat liver nuclei^a^.

		Δ*H*_exc_				
Treatment		Total	I	Additional	II	III
Buffer A (polyamines)						
	Control	24.7	4.1 (17%)	1.0 (4%)	11.7 (47%)	7.6 (31%)
+Dst		34.5	2.6 (7.5%)	2.8 (8%)	10.4 (30%)	17.6 (51%)
Buffer B (Mg^2+^)						
	Control	35.8			31.5 (88%)	3.9 (11%)
+Dst		33.7	2.7 (8%)		24.5 (73%)	6.0 (18%)

a Δ*H*_exc_ are expressed in kJ/mol nucleotides of DNA and in the percentage of total Δ*H*_exc_. The results represent the mean of at least three experiments with standard deviation from mean not exceeded 5%.

At incubation of nuclei with Dst the antibiotic enters into the nuclei and forms a complex with nuclear DNA, which is easily observed by means of the appearance of the signal at *λ* = 320 nm in the CD spectrum (Prusov et al. 2010).

The interaction of antibiotic with nuclei at molar ratio Dst/DNA from 0.01 to 0.1 in both buffer systems caused the concentration-dependent changes in endotherm parameters. At the molar ratio Dst/DNA = 0.1 (the saturation ratio for naked DNA at 1:1 binding mode, Schultz & Dervan 1984), the effects were most pronounced. Represented scans of nuclei up to 120 °C in buffers A and B at Dst concentration 0.15 mM (molar ratio DM/DNA = 0.1) are shown in Figures 1 and 2 (curve 2).

In buffer A, we observed the drift of peaks II and III at 89° and 101° to 95° and 107 °C, respectively (Figure 1, curve 2). Low-temperature peak I (75 °C), on the contrary, shifted to *T*m = 73 °C which is lower compared with the control. It should be noted the appearance of a broad plateau-like intermediate peak between 77 and 87 °C. At the same time, total Δ*H*_exc_ is increased by about 1.5 times. The ratio of the areas under the peaks altered also (see Table 1). Peak III becomes dominant, while peak II remains almost unchanged. Δ*H*_exc_ of the low-temperature peak I decrease.

After incubation of nuclei with Dst, the thermogram of nuclei in buffer B becomes like that of nuclei in buffer A and also reveals three peaks with the transition temperatures at 77, 91 and 100 °C. We suggested that in nuclei in buffer B domains I and II were fused because *T*m's were close. The appearance of domains with *T*m higher and lower than these in the control nuclei indicates that Dst destabilizes the domain of core-histone and strengthens DNA domains. However, the values of total Δ*H*_exc_ in contrast to the melting of nuclei in buffer A do not change significantly.

Based on the data listed in Table 1, we calculated that at subtraction of the Δ*H*_exc_ of manifested endotherm I (77 °C) which appeared from main (I + II) endotherm of control scan (−8%) relative decreasing Δ*H*_exc_ of peak II (from 80% to 73%) and increasing Δ*H*_exc_ of peak III (from 11% to 18%) in the Dst presence are similar and equal approximately to 7%. It is possible that a part of the material of peak II passed in peak III. Other cause of Δ*H*_exc_ permanence in buffer with Mg^2+ ^may be retention of Dst on only one strand of DNA (Wan et al. 2000; David et al. 2002) and therefore does not require additional heat to break the DNA–Dst interaction.

It should be noted that because of some uncertainty in drawing of baseline and sensitivity of ‘null – balance’ instruments to subtle factors the enthalpy changes calculated from each peak must be considered as only approximate. However, they definitely characterize the trends of the peaks.

Thus, both the buffer systems (cell nuclei in buffers A and B) may be used to test drugs by DSC and two main features – increasing of Tm of high-temperature peaks (shift right) and decreasing of Tm of low-temperature peak (shift left) – may serve as a basis for the classification of oligopyrrole minor groove binders antibiotics. So, this approach can be exploited as a drug screening of Dst derivatives in cisplatin-sensitive and -resistant ovarian cancer cells (Marverti et al. 2012).

## Discussion

DSC method allows ‘to fractionate’ nuclear chromatin to specific independent energy domains. Differences between thermograms of rat liver nuclei isolated in the presence of multi-charge physiologically significant cations – spermine and spermidine – or ions Mg^2+ ^indicate the important and different roles of these cations in 3D chromatin structure and its energy domains formation (Visvanathan et al. 2013). The isolation of cell nuclei with compact chromatin structure in the presence of cations is a standard method. We used the buffers A and B containing 0.2 mM spermine and 0.5 mM spermidine (Gasser & Laemmli 1987) or 5 mM Mg^2+^, respectively. These values are close to those indicated in the work by Visvanathan et al. (2013). Authors showed that the compaction state within cell nuclei *in vivo* is strongly influenced by the levels of Ca^2+^, Mg^2+^ and polyamines. The maximum compactness was observed at 6–8 mM Ca^2+^ or Mg^2+^ and at 1.5 mM spermidine and 0.4 mM spermine. The concentrations of cations associated with chromatin within the nucleus are unknown but in employed by us cations environment the native compact chromatin structure is supported at isolation of nuclei. In our experiments the compaction state of chromatin was verified by electron microscopy. The observed differences in the parameters of the nuclei thermograms in the applied buffers may be related to particular features of interfibrillar bridges formed by polyanions or Mg^2+^ ions. Another reason for changes in the thermograms, apparently, is the partial DNA fragmentation by endogenous nucleases in the presence of Mg^2+^ ions. It is reflected in the reduction of the peak III, sensitive to nuclease action (Rice et al 1988; Almagor & Cole 1989a). In our experiments, a similar situation may occur at the initial moment of homogenization of tissue even in the presence of a nuclease inhibitor NEM. However, electrophoretic control showed that after purification through sucrose with high density in the presence of NEM the bulk of DNA remains highly polymeric, including long-term storage at −60 °C (not illustrated). Herewith, the chromatin in the nucleus retains the original condensed state.

Nuclei in both used systems for melting (buffer A and B) were sensitive to the action of minor groove binder antibiotic Dst. The altered thermal profile observed in the presence of Dst distinguishes from that induced in nuclei by other drugs such as bleomycin, streptonigrin, methylnitrosourea, mitomycin and ultraviolet light. DSC scan of nuclei in the presence of Dst is more similar to that observed for the thermograms under action on nuclear chromatin of intercalating dyes (Almagor & Cole 1989b). The action of Dst on nuclei results in the appearance of high-temperature peaks II and III with *T*m higher than *T*m in control thermogram, whereas single and double strand breaks of DNA usually decrease *T*m of high-temperature peaks (Almagor & Cole 1989b). We assume that Dst stabilizes DNA of nuclear chromatin possibly due to the formation additive of bifurcate hydrogen bonds and hydrophobic interactions of Dst between the both DNA chains (Coll et al. 1987). At the same time, the important consequence of Dst action is remarkable destabilization of the interactions between DNA and histones that is revealed in the decreasing of *T*m and Δ*H* of low-temperature peak I on DSC-thermograms.

It is seen from the Table 1 data that the binding of Dst to different domains of nuclear chromatin is not identical. The largest increase in Δ*H*_exc_ occurs at the peak III, which we attributed to constrained DNA. It is possible that antibiotic prefers to associate with topologically constrained DNA. Otherwise, the action of Dst on nuclear chromatin may result in conformational transitions of DNA (Dolenc et al. 2005; Lah et al. 2008). It is to be exemplified by nuclear melting in buffer B when the increasing of Δ*H*_exc_ of peak III was accompanied by the equally decrease of peak II. On the other hand, our calorimetric investigation did not allow us to discard the assumption on the diverse manners of Dst binding in the media with different multivalent cations (polyamine and Mg^2+^). Thus, one may suggest that therapeutic effect of Dst has come from a more strong binding between chains of DNA thereby complicating the work of the enzymes on the DNA template (Pushcendorf et al. 1971) and the weakening of protein interaction with DNA.

## Conclusions

We tested for the first time the binding of oligopyrrole drug directly within isolated cell nuclei by DSC method suitable for the study of turbid media. Our results contribute towards validation of DSC to test drugs ability to modulate chromatin structure in the physiological environment and to clarify the mechanism of these modulations. We found that Dst strengthens interaction between DNA chains and weakens histones-DNA contacts upon binding the drug with chromatin within a nucleus. One can speculate that performed study has a potential in perspective to demonstrate the relations between the energetic parameters of the drug binding and the *T*m shifts in thermogram of nuclear chromatin.
